# Sinomenine Protects Against Morphine Dependence through the NMDAR1/CAMKII/CREB Pathway: A Possible Role of Astrocyte-Derived Exosomes

**DOI:** 10.3390/molecules23092370

**Published:** 2018-09-17

**Authors:** Jinying Ou, Yuting Zhou, Chan Li, Zhijie Chen, Hancheng Li, Miao Fang, Chen Zhu, Chuying Huo, Ken Kin-Lam Yung, Jing Li, Chaohua Luo, Zhixian Mo

**Affiliations:** 1 School of Traditional Chinese Medicine, Southern Medical University, 1023-1063 Shatai South Road, Guangzhou 510515, China; ojyresearch@163.com (J.O.); zytingkv@icloud.com (Y.Z.); lichan1874@163.com (C.L.); chen15625158269@outlook.com (Z.C.); lihancheng0601@126.com (H.L.); 13268268224@163.com (M.F.); 18820795251@163.com (C.Z.); fokchorying@163.com (C.H.); 2 Department of Biology, Hong Kong Baptist University, Kowloon Tong, Hong Kong, China; kklyung@hkbu.edu.hk; 3 Central Laboratory, Southern Medical University, 1023-1063 Shatai South Road, Guangzhou 510515, China; lijing.hs@163.com

**Keywords:** sinomenine, morphine, exosomes, NMDAR1, CAMKII, CREB

## Abstract

Sinomenine is a nonaddictive alkaloid used to prevent morphine dependence, even thoughits mechanism isnot fully understood. Astrocytes aggravate the pathological process in their neighboring cellsthrough exosomes in central nervous system diseases. However, the effect of sinomenine on astrocyte-derived exosomes for the amelioration of morphine dependence has not been reported yet. In this study, we found that sinomenine prevented the morphine-induced conditionedplace preference in mice. Sinomenine reduced the levels of cAMP and intracellular Ca^2+^ in morphine-treated SH-SY5Y cells. Moreover, sinomenine inhibited the expressions of p-NMDAR1/NMDAR1, p-CAMKII/CAMKII, and p-CREB/CREB in the hippocampusof morphine-dependent mice and SH-SY5Y cells. Furthermore, we found that sinomenine inhibitedthe morphine-induced activation of astrocytesin vivo and in vitro. Afterwards, exosomes were isolated from cultured primary astrocytes treated with phosphate buffer saline (PBS, ctl-exo), morphine (mor-exo), or morphine and sinomenine (Sino-exo). Subsequently, morphine-treated SH-SY5Y cells were treated with ctl-exo, mor-exo, and Sino-exo. Results showed that Sino-exo reduced the level of cAMP, intracellular Ca^2+^, and the expression of p-CAMKII/CAMKII and p-CREB/CREB in morphine-treated SH-SY5Y cells. In conclusion, we demonstrated that sinomenine exhibited protective effects against morphine dependencein vivo and in vitro through theNMDAR1/CAMKII/CREB pathway. Sinomenine-induced alterationof the function of astrocyte-derived exosomes may contribute to the antidependence effects of sinomenine in morphine dependence.

## 1. Introduction

Drug dependence is considered as an aberrant form of learning and memory, generating strong associations that link action to drug seeking, increasing the vulnerability to relapse [1,2]. Therefore, it is crucial to reduce drug addiction-induced memory to prevent morphine dependence. The hippocampus is an important brain region for the formation of addiction memory [3]. Previous studies have found that long-term potentiation (LTP) and long-term depression (LTD) of postsynaptic membranes of glutamatergic neurons in the hippocampus are involved in drug addiction-related behaviors, such as relapse and behavioral sensitization [4]. N-methyl-d-aspartate (NMDA) receptors, one of the excitatory glutamate receptors, take part in the formation of LTP processes in the postsynaptic membrane [5]. Chronic morphine administration stimulates the phosphorylation of NR1 Ser897, leading to the opening of ion channels, which allows Ca^2+^ influx. The increased intracellular Ca^2+^ further activates CAMKII and initiates a series of kinase cascade signaling pathways, which cause CREB phosphorylation [6]. CREB mediates the transcription of target genes and ultimately forms the reward memories for drugs [7]. Therefore, the NMDAR1/CAMKII/CREB signaling pathway in the hippocampus may play a crucial role in the formation of addiction memory.

Sinomenine is an active ingredient extracted from *Sinomeniumacutum*, a Chinese traditional medicinal plant [8]. Sinomenine does not exhibit addictive potency itself, though its chemical structure is similar to that of morphine [9]. Our previous research has included several experimental and clinical studies on the prevention and treatment of morphine dependence using sinomenine [10,11,12]. These studies showed that sinomenine has therapeutic effects on the withdrawal symptoms of physical dependence as well as psychological dependence in addicted animals. However, the underlying mechanisms have not been fully defined.

Astrocytes are the most abundant and generally distributed cell type in the mammalian brain. Astrocytes regulate biological processes in neurons and other glial cells by releasing glutamate [13], lactate [14], D-serine [15], γ-aminobutyric acid (GABA), adenosine triphosphate, and taurine [16]. Furthermore, glial cell-line-derived neurotrophic factor (GDNF), brain-derived neurotrophic factor (BDNF), and basic fibroblast growth factor (bFGF), secreted by astrocytes, have been reported to be closely related to drug addiction [17]. These studies suggested that astrocytes might play an important role in supporting and regulating the long-term maintenance of drug addiction.

Exosomes are nanoscale vesicles ranging from 30 to 150 nm in diameter with a lipid bilayer membrane structure. Almost all the cells in the central nervous system (CNS) can secrete exosomes, including neural stem/progenitor cells, neuronal cells, microglia, astrocytes, and oligodendrocytes [18]. In the CNS, exosomes mediate intercellular communication of adjacent or distant neurons, which regulates neural development, differentiation, regeneration, and the plasticity of neuronal glutamatergic synapses [19]. Hu et al. reported that astrocytes treated with morphine and human immunodeficiency virus (HIV) Tat increased the expression of miR-29b released by exosomes; exosomal miR-29b was subsequently taken up by neurons, where it downregulated the expression of PDGF-B, thus accelerating the HIV Tat and morphine-mediated neuronal dysfunction [20]. This study suggested that astrocyte-derived exosomes might impact the pathological process of morphine dependence. However, this new insight into morphine dependence has not yet been fully clarified and is the research target of this study.

In this study, we aimed to investigate the effect of sinomenine on morphine dependence through the NMDAR1/CAMKII/CREB signaling pathway in vitro and in vivo. We then observed the changes in astrocytes in the process of morphine dependence and analyzed their relationship with addiction. Meanwhile, the exosomes secreted by astrocytes were extracted to study their biological characteristics and the effects on morphine dependencein vitro.

## 2. Results

### 2.1. Sinomenine Inhibited Morphine-Induced Conditioned Place Preference in Mice

**Figure 1:**
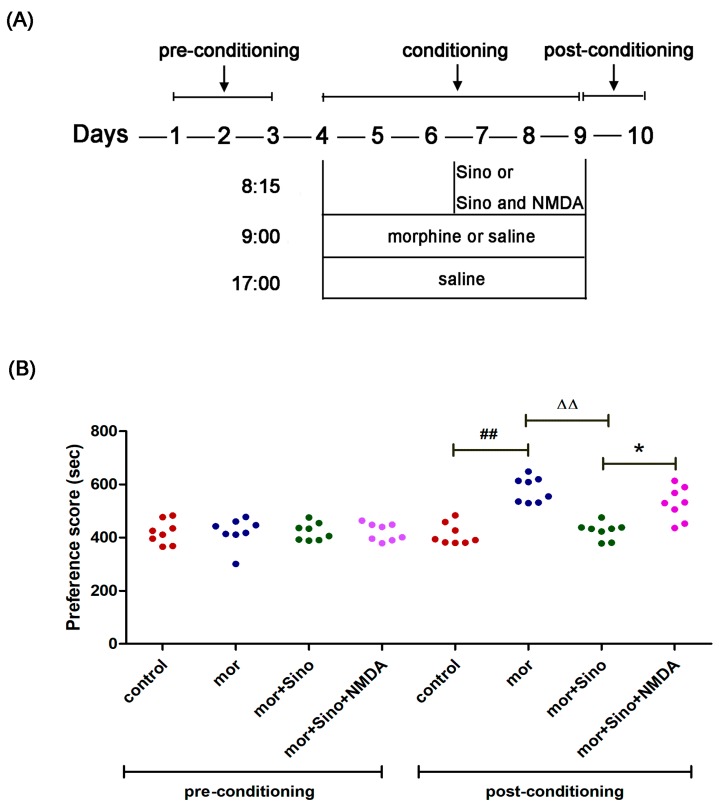
Sinomenine inhibited morphine-induced conditioned place preference in mice. (**A**) Schematic protocol of the conditioned place preference testing; (**B**) The time spent in the white compartment in preconditioning and postconditioning phases (*n* = 8). ^##^ *p* < 0.01 vs. control, ^ΔΔ^ *p* < 0.01 vs. mor, * *p* < 0.05 vs. mor + Sino. Mor, morphine; Sino, sinomenine; NMDA, *n*-methyl-d-aspartate.

Conditioned place preference is the classic experiment for evaluating drug dependence [21]. As shown in Figure 1B, in the preconditioning phase, there was no significant difference in time spent in the white compartment among all groups (*p* > 0.05). After morphine administration and training for 6 consecutive days, the mice in the morphine group significantly spent more time in the white compartment than the control group (*p* < 0.01). The group that received sinomenine pretreatment spent significantly shorter time in the white compartment compared with the morphine group (*p* < 0.01). The inhibitory effect of sinomenine on conditioned place preference was encountered by NMDA receptor agonists, as showed by the mor + Sino + NMDA group. These results implied that the inhibitory effect of sinomenine on morphine dependence might be associated with NMDA receptors.

### 2.2. Sinomenine Inhibited the Expression of p-NMDAR1/NMDAR1, p-CAMKII/CAMKII, and p-CREB/CREB in the Hippocampus of Morphine-Dependent Mice

**Figure 2:**
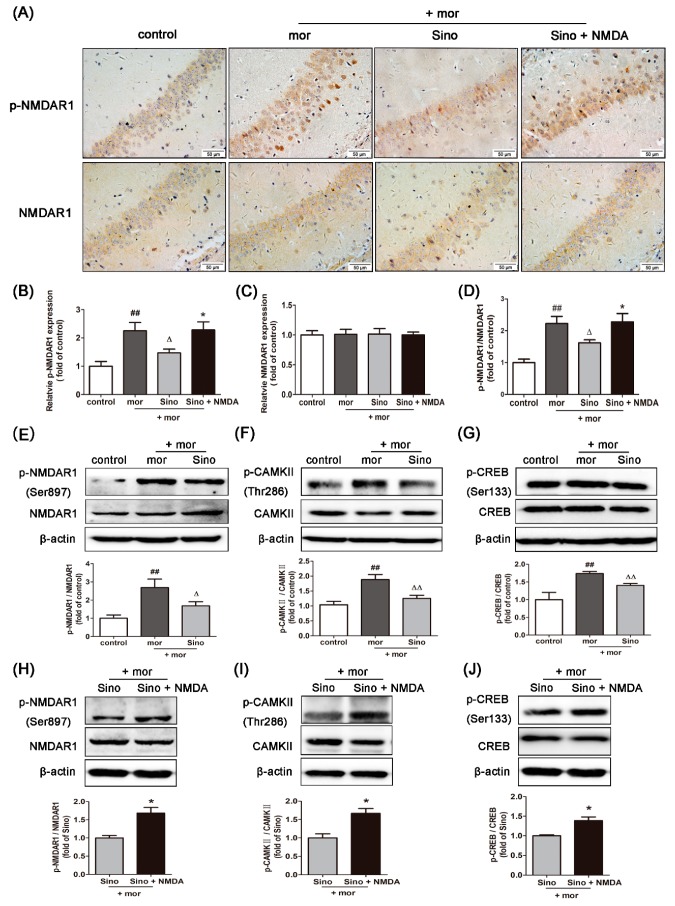
Sinomenine inhibited the expressions of p-NMDAR1/NMDAR1, p-CAMKII/CAMKII, and p-CREB/CREB in the hippocampus of morphine-dependent mice. (**A**–**D**) Immunohistochemistry analysis was used to quantify the expression levels of p-NMDAR1 and NMDAR1 in the hippocampus of morphine-dependent mice (*n* = 3); (**E**–**J**) Western blotting analysis was used to quantify the expression levels of p-NMDAR1/NMDAR1, p-CAMKII/CAMKII, and p-CREB/CREB in the hippocampus (*n* = 3). ^##^ *p* < 0.01 vs. control; ^Δ^ *p* < 0.05, ^ΔΔ^ *p* < 0.01 vs. mor; * *p* < 0.05 vs. Sino.

Compared with that in the control group, the expression of p-NMDAR1 was significantly increased in the morphine group (Figure 2A,B, *p* < 0.01). Sinomenine pretreatment significantly decreased the expression of p-NMDAR1 (Figure 2A,C, *p* < 0.01). However, there was no significant difference in the expression of NMDAR1 in the hippocampus among all groups (Figure 2A,C, *p* > 0.05). Nevertheless, compared with that in the control group, the ratio of p-NMDAR1/NMDAR1 in the morphine group was significantly increased, indicating the activation of NMDAR1 in the hippocampus of morphine-dependent mice (Figure 2D, *p* < 0.01). Pretreatment with sinomenine significantly decreased the ratio of p-NMDAR1/NMDAR1 in the mor + Sino group compared with that in the morphine group (Figure 2D, *p* < 0.01). These results are consistent with the results from Western blotting analysis (Figure 2E).

We further analyzed the downstream proteins of NMDAR1-CAMKII and CREB. Compared to the control group, the ratio of p-CAMKII/CAMKII and p-CREB/CREB was significantly increased in the hippocampus of mice in the morphine group (Figure 2F,G, *p* < 0.01). Sinomenine pretreatment significantly decreased the ratio of p-CAMKII/CAMKII and p-CREB/CREB in the mor + Sino group compared with that in the morphine group (Figure 2F,G, *p* < 0.01), indicating that sinomenine inhibited the activation of CAMKII and CREB in the hippocampus of morphine-dependent mice. These inhibitory effects of sinomenine on p-NMDAR1/NMDAR1, p-CAMKII/CAMKII, and p-CREB/CREB could be significantly reversed by NMDA receptor agonists (Figure 2H–J, *p* < 0.05).

### 2.3. Sinomenine Decreased the Level of cAMP in Morphine-Treated SH-SY5Y Cells

**Figure 3:**
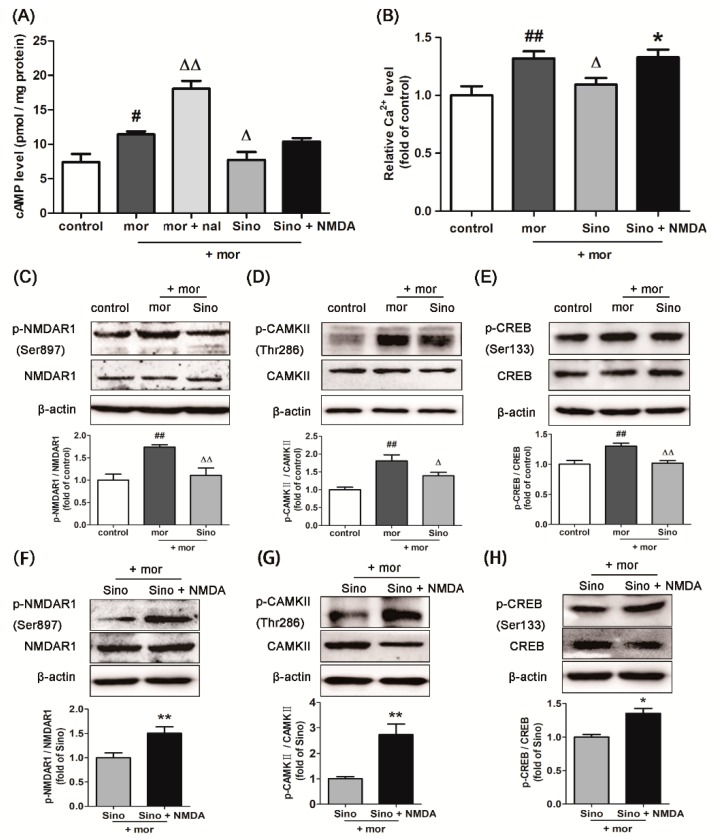
Intervention of sinomenine on morphine-treated SH-SY5Y cells. (**A**) Sinomenine decreased the level of cAMP in the morphine-treated SH-SY5Y cells (*n* = 3). SH-SY5Y cells were pretreated with 100 μMsinomenine for 12 h and then incubated with 100 μM morphine for 48 h. NMDAR agonist was incubated with SH-SY5Y cells treated with morphine and sinomenine for 12 h. Naloxone was incubated with SH-SY5Y cells treated with morphine to allow withdrawal for 20 min before evaluation. mor: morphine; nal: naloxone; (**B**) Sinomenine decreased the level of intracellular Ca^2+^ in the morphine-treated SH-SY5Y cells (*n* = 3); (**C**–**E**) Sinomenine suppressed the expression of p-NMDAR1/NMDAR1, p-CAMKII/CAMKII, and p-CREB/CREB, as detected by Western blotting in the morphine-treated SH-SY5Y cells (*n* = 3); (**F**–**H**) NMDAR agonist reversed the inhibitory effect of sinomenine on p-NMDAR1/NMDAR1, p-CAMKII/CAMKII, and p-CREB/CREB (*n* = 3). ^#^ *p* < 0.05, ^##^ *p* < 0.01 vs. control; ^Δ^ *p* < 0.05, ^ΔΔ^ *p* < 0.01 vs. morphine; * *p* < 0.05, ** *p* < 0.01 vs. Sino.

Chronic morphine induced a relatively high level of cAMP in the neurons of drug-dependent mice. When opiate receptor antagonists were used to prompt withdrawal, the level of intracellular cAMP raised sharply. This occurrence is known as cAMP overshoot. This phenomenon is considered one of the key indicators of the establishment of a morphine-dependence-like cell model [22]. As shown in Figure 3A, compared with that in the control group, morphine treatment for 48 h (100 μM, Figure S2A in the Supplementary Materials) significantly increased the level of intracellular cAMP in SH-SY5Y cells (*p* < 0.05). After treatment by 100 μΜ naloxone, the level of intracellular cAMP in SH-SY5Y cells was significantly increased compared with that in the morphine group (*p* < 0.01). This result indicated that a group of morphine-dependence-like SH-SY5Y cells was successfully established. Pretreatment with sinomenine (100 μM, Figure S2B in the Supplementary Materials) significantly decreased the level of cAMP in morphine-treated SH-SY5Y cells (*p* < 0.05). Although NMDA receptor agonist induced a higher level of intracellular cAMP level than that of the sinomenine group, the difference was not significant (*p* > 0.05).

### 2.4. Sinomenine Decreasedthe Level of Intracellular Ca^2+^ in Morphine-Treated SH-SY5Y Cells

As shown in Figure 3B, compared with that in the control group, 100 μM morphine treatment for 48 h significantly increased the level of intracellular Ca^2+^ in SH-SY5Y cells (*p* < 0.01). Pretreatment of 100 μM sinomenine significantly decreased the level of Ca^2+^ in morphine-treated SH-SY5Y cells compared with the morphine group (*p* < 0.05). This inhibitory effect of sinomenine on intracellular Ca^2+^ was significantly reversed by NMDA receptor agonists (*p* < 0.05).

### 2.5. Sinomenine Inhibitedthe Expressions of p-NMDAR1/NMDAR1, p-CAMKII/CAMKII, and p-CREB/CREB in Morphine-Treated SH-SY5Y Cells

As shown in Figure 3C–E, compared with that in the control group, 100 μM morphine treatment for 48 h significantly increased the ratio of p-NMDAR1/NMDAR1, p-CAMKII/CAMKII, and p-CREB/CREB in SH-SY5Y cells (*p* < 0.01). Pretreatment of 100 μM sinomenine significantly decreased the ratio of p-NMDAR1/NMDAR1 (*p* < 0.01), p-CAMKII/CAMKII (*p* < 0.05), and p-CREB/CREB (*p* < 0.05) compared with the morphine group. These inhibitory effects of sinomenine on p-NMDAR1/NMDAR1, p-CAMKII/CAMKII, and p-CREB/CREB were significantly reversed by NMDA receptor agonists (Figure 3F–H, *p* < 0.01, *p* < 0.01, *p* < 0.05).

### 2.6. Sinomenine Inhibited the Activation of Astrocytes Induced by Morphine

**Figure 4:**
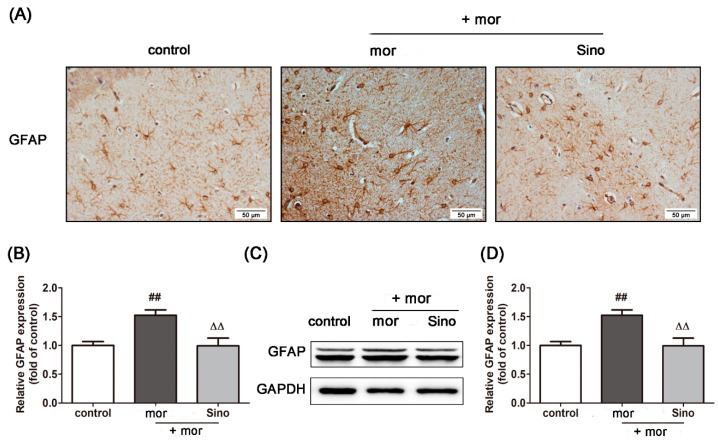
Sinomenine inhibited the activation of morphine-treated astrocytes. (**A**,**B**) Immunohistochemistry analysis was used to quantify the expression level of GFAP in the hippocampus of morphine-dependent mice (*n* = 3); (**C**,**D**) Western blotting analysis was used to quantify the expression level of GFAP in cultured primary astrocytes (*n* = 3). Cultured primary astrocytes were pretreated with phosphate buffer saline (PBS) or 200 μM sinomenine for 12 h and then incubated with 100 μM morphine for 48 h. ^##^ *p*< 0.01 vs. control; ^ΔΔ^ *p* < 0.01 vs. morphine.

As shown in Figure 4A,B, compared with that in the control group, the expression of GFAP in the hippocampus of morphine-dependent mice was significantly higher, indicating the activation of astrocytes (Figure 4A,B, *p* < 0.01). Pretreatment of sinomenine significantly decreased the expression of GFAP compared with the morphine group (*p* < 0.01). A consistent result was observed in cultured primary astrocytes. Morphine treatment for 48 h (100 μM, Figure S2C in the Supplementary Materials) significantly increased the expression of GFAP in cultured primary astrocytes. Pretreatment with sinomenine (200 μM, see Figure S2D in the Supplementary Materials) significantly inhibited the expression of GFAP in primary astrocytes treated with morphine relative to the expression in the morphine group (Figure 4C,D, *p* < 0.01).

### 2.7. Astrocyte-Derived Exosomes were Identified byTransmission Electron Microscope, Nanoparticle Size Analysis, and Marker Protein

**Figure 5:**
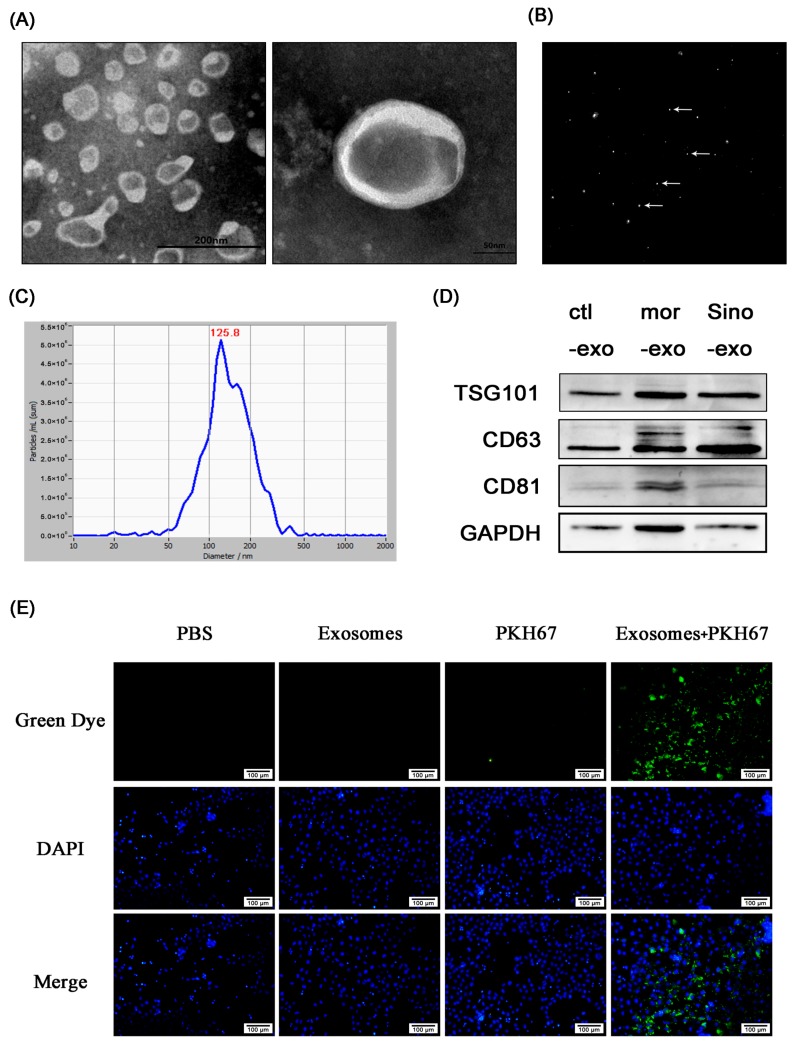
Astrocyte-derived exosomes can be taken up by neuronal SH-SY5Y cells. (**A**) Exosomes observed by transmission electron microscope; (**B**,**C**) Nanoparticle tracking analysis of exosomes; (**B**) The signal of scattered light by dynamic nanoparticles; the bright white dot indicates one moving particle; (**C**) Particle size distribution of nanoparticles; (**D**) Western blotting was used to detect TSG101, CD63, andCD81 proteins of exosomes. Ctl-exo: exosomes extracted from cultured primary astrocytes; mor-exo: exosomes extracted from cultured primary astrocytes treated with 100 μM morphine for 48 h; Sino-exo: exosomes extracted from cultured primary astrocytes treated with 100 μM morphine and 200 μMsinomenine for 48 h; (**E**) Astrocyte-derived exosomes were labeled by PKH67 and were taken up by neuronal SH-SY5Y cells.

Transmission electron microscopy showed that the isolated exosomes were round or ovoid in shape with a lipid bilayer membrane structure, and ranged from 20–100 nm in diameter (Figure 5A). Nanoparticle tracking analysis showed that the size of the exosomes was around 125.8 nm (Figure 5B,C). The extracted exosomes were further analyzed by Western blotting. Exosome marker proteins TSG101, CD63, and CD81 were found in the exosomes secreted by astrocytes after treatment with morphine and sinomenine (Figure 5D).

### 2.8. Cultured Primary Astrocyte-Derived Exosomes Can Be TakenUp by Neuronal SH-SY5Y Cells

Exosomes labeled with PKH67 were incubated with neuronal SH-SY5Y cells for 12 h. As shown in Figure 5E, a large number of spot-like green fluorescence puncta were observed in the cytoplasm of PKH67-labeled exosomes, with little residue of fluorescence dye. This result indicated that cultured primary astrocyte-derived exosomes can be phagocytosed and internalized by neuronal SH-SY5Y cells.

### 2.9. Effect of Astrocyte-Derived Exosomes on cAMP Level in Morphine-Treated SH-SY5Y Cells and Subsequent Intervention by Sinomenine

**Figure 6:**
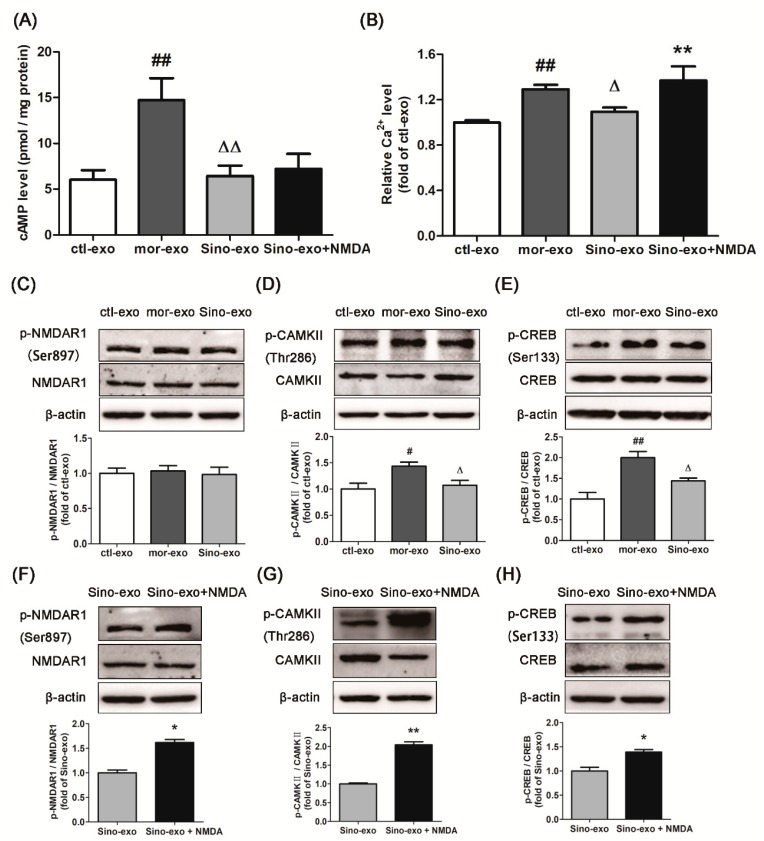
Effects of astrocyte-derived exosomes on morphine-treated SH-SY5Y cells and subsequent intervention by sinomenine. (**A**) Sino-exo decreased the level of cAMP induced by mor-exo in the morphine-treated SH-SY5Y cells (*n* = 3); (**B**) Sino-exodecreased the level of intracellular Ca^2+^ induced by mor-exo in the morphine-treated SH-SY5Y cells (*n* = 3); (**C**–**E**) Effects of Sino-exo on p-NMDAR1/NMDAR1, p-CAMKII/CAMKII, and p-CREB/CREB induced by mor-exo in the morphine-treated SH-SY5Y cells (*n* = 3); (**F**–**H**) NMDAR agonist reversed the inhibitory effect of Sino-exo on p-NMDAR1/NMDAR1, p-CAMKII/CAMKII, and p-CREB/CREB (*n* = 3). Ctl-exo: exosomes extracted from cultured primary astrocytes; mor-exo: exosomes extracted from cultured primary astrocytes treated with 100 μM morphine for 48 h; Sino-exo: exosomes extracted from cultured primary astrocytes treated with 100 μM morphine and 200 μMsinomenine for 48 h. ^#^ *p* < 0.05, ^##^ *p* < 0.01 vs. ctl-exo; ^Δ^ *p* < 0.05, ^ΔΔ^ *p* < 0.01 vs. mor-exo; * *p* < 0.05, ** *p* < 0.01 vs. Sino-exo.

After SH-SY5Y cells were treated with 100 μM morphine for 24 h, 3 g/mL exosomes secreted by cultured primary astrocyte cells (ctl-exo), morphine-treated (mor-exo), or morphine + sinomenine-treated astrocyte cells (Sino-exo) were added to the cells, which were further incubated with 100 μM morphine for 24 h. The level of cAMP was determined. As shown in Figure 6A, the level of cAMP in the mor-exo group was significantly higher than that in the ctl-exo group (*p* < 0.01). This result indicated that astrocyte-derived exosomes with chronic morphine treatment could further increase cAMP levels in morphine-treated SH-SY5Y cells. Compared to that in the mor-exo group, the level of cAMP in the Sino-exo group was significantly lower (*p* < 0.01). The cAMP level was upregulated upon treatment with Sino-exo and the NMDA receptor agonist, but this level was not significantly different from that in the Sino-exo group (*p* > 0.05).

### 2.10. Effects of Astrocyte-Derived Exosomes on the Level of Intracellular Ca^2+^ in Morphine-Treated SH-SY5Y Cells and Subsequent Intervention by Sinomenine

After SH-SY5Y cells were treated with 100 μM morphine for 24 h, 6μg/mL exosomes secreted by cultured primary astrocyte cells (ctl-exo), or morphine-treated (mor-exo), or morphine + sinomenine-treated astrocyte cells (Sino-exo) were added to the cells, which were then incubated with 100 μM morphine for another 24 h. The level of intracellular Ca^2+^ was evaluated. As shown in Figure 6B, compared with that in the ctl-exo group, the level of intracellular Ca^2+^ in the mor-exo group was significantly higher (*p* < 0.01). This result indicated that astrocyte-derived exosomes with chronic morphine treatment could further increase the intracellular Ca^2+^ level in morphine-treated SH-SY5Y cells. Compared with that in the mor-exo group, the level of intracellular Ca^2+^ in the Sino-exo group was significantly lower (*p* < 0.01). Cells incubated with Sino-exo and NMDA receptor agonist showed significantly higher levels of intracellular Ca^2+^ than the Sino-exo group (*p* < 0.01).

### 2.11. Effects of Astrocyte-Derived Exosomes on the Expression of p-NMDAR1/NMDAR1, p-CAMKII/CAMKII, and p-CREB/CREB in Morphine-Treated SH-SY5Y Cells and Subsequent Intervention by Sinomenine

The cells were incubated with exosomes according to the same protocol as described in Section 2.10. As shown in Figure 6C, there was no significant difference in the expression of p-NMDAR1/NMDAR1 among three groups. Compared with the ctl-exo, mor-exo showed significantly higher ratio of p-CAMKII/CAMKII (Figure 6D, *p* < 0.05) and p-CREB/CREB (Figure 6E, *p* < 0.01) in morphine-treated SH-SY5Y cells. Compared with the mor-exo group, Sino-exo inhibited the expression of p-CAMKII/CAMKII (Figure 6D, *p* < 0.05) and p-CREB/CREB (Figure 6E, *p* < 0.05). The inhibitory effect of Sino-exo on p-CAMKII/CAMKII and p-CREB/CREB in the morphine-treated SH-SY5Y cells could be reversed by the NMDA receptor agonist (Figure 6G,H, *p* < 0.01, *p* < 0.05).

## 3. Discussion

Drug dependence is a troublesome issue that perplexes the fields of medicine and public health. However, the effect of current therapies such as drug, psychological, and social interventions are still unsatisfactory [23,24]. Traditional Chinese medicine has been used to treat drug dependence for more than 200 years. Modern clinical research and animal experiments have found that traditional Chinese medicine has a curative effect in preventing drug relapse, relieving physical dependence and withdrawal symptoms [25]. Sinomenine is the main alkaloid found in *Sinomeniumacutum*, a traditional Chinese herbal medicine. In this study, we found that sinomenine not only prevented the conditioned place preference (CPP) induced by morphine in vivo, but also reduced morphine-dependence-like effects in vitro. Its mechanism may be related to the downregulation of the NMDAR1/CAMKII/CREB memory formation pathway.

In morphine-dependent mice and morphine-treated cells, the morphine-induced mental dependence effect was related to the upregulation of the NMDAR1/CAMKII/CREB memory formation pathway. Previous studies showed that addictive drugs stimulated the presynaptic membrane to release glutamate, which led to the rapid activation of α-amino-3-hydroxy-5-methyl-4-isoxazole-propionic acid-type glutamate receptors (AMPARs) and depolarization in the postsynaptic membrane; as a consequence, NMDA receptors were activated after the sufficient depolarization mediated by AMPARs [26]. Extracellular calcium influx through activated NMDA receptors subsequently activates the downstream protein kinases of NMDA receptors and memory nuclear transcription factors, which regulate the expression of target genes and form new synapses, leading to the consolidation of addiction memory [27]. Siahposhtkhachaki et al. found that the preadministration of AP5, which can block NMDA receptors, reduces the ratio of p-CREB/CREB in the hippocampus and promotes the regression of conditioned place preference in rats [28]. These studies suggest that the formation and consolidation of morphine addiction memory is related to NMDA receptors. Therefore, we observed the effect of sinomenine on NMDA receptors. For the first time, we found that sinomenine inhibits the activation of NMDAR1 receptors in the mouse hippocampus and in morphine-treated SH-SY5Y cells, though this effect could be counteracted by NMDA receptor agonists. After the administration of NMDA receptor agonists, preference for the drug reward conditioning location was obviously increased in the morphine-dependent mice pretreated with sinomenine. Similarly, NMDA receptor agonist activated NMDAR1 and increased the level of cAMP in morphine-treated SH-SY5Y cells pretreated with sinomenine. These results suggested that the activation of NMDAR1 might contribute to the reward effects and the formation of morphine-induced mental dependence. The inhibition effect of sinomenine on morphine mental dependence might be related to the inhibition of NMDAR1 activation.

In opioid tolerance and addiction, NMDA receptors and their downstream proteins, such as CAMKII and CREB, are involved in the formation of long-term memory. In morphine dependence, the level of intracellular Ca^2+^ and activity of the protein downstream to NMDAR1, CAMKII, are increased due to the activation of NMDAR1 receptors; following the activation of NMDAR1 receptors, the opening of ion channels leads to the influx of the Ca^2+^ in the postsynaptic membrane, and the CAM/Ca^2+^ complex induces the phosphorylation of multiple serine and threonine sites in CAMKII [29]. Our results are consistent with these previous studies. Activated CAMKII further activates the transcription factors, such as CREB. Phosphorylated CREB initiates a series of downstream responses that play a significant role in opioid addiction [30], withdrawal [31], and relapse [32]. Opioid addiction is a strong, long-lasting memory with synaptic long-term potentiation (LTP) as its electrophysiological basis. In LTP, CREB promotes the expression of LTP-related genes and generates the protein needed for memory consolidation [33]. In this study, we observed that sinomenine inhibited the activation of NMDAR1 and its downstream proteins, CAMKII and CREB, in the hippocampus of morphine-dependent mice and in SH-SY5Y cells. The underlying mechanism might be through the inhibition of NMDAR1 activation and reduction of the opening of calcium channels to decrease the influx of intracellular Ca^2+^, which then inhibited the phosphorylation of CAMKII and activation of CREB.

The present study found that chronic exposure to morphine activated astrocytes, which is consistent with previous studies [34]. Narita reported that the injection of astrocyte-conditioned medium (ACM) into the nucleus accumbens increased the CPP score of mice and increased the reward effect of morphine and methamphetamine; single-cell chemical attractant protein-5 (MCP-5) and soluble tumor necrosis factor receptor 1 (sTNFR1) from ACM may contribute to this reward effect [35]. Furthermore, Arezoomandan et al. reported that the injection of conditioned medium from methamphetamine-treated astrocytes into the bilateral nucleus accumbens prolonged the regression of CPP in rats, suggesting that the activated astrocytes may be involved in the maintenance of methamphetamine-induced addiction [36]. These studies showed that astrocytes may indirectly participate in the maintenance of drug dependence by secreting soluble factors in certain brain regions. Therefore, we extracted exosomes from primary astrocytes cultured in a conditioned medium and found that astrocyte-derived exosomes were taken up by neuronal SH-SY5Y cells. This uptake may be the first step for astrocyte-derived exosomes to regulate the biological process of the recipient cells.

In order to determine the role of astrocyte-derived exosomes in morphine dependence, astrocyte-derived exosomes were administered to the morphine-treated SH-SY5Y cells. Compared to control astrocyte-derived exosomes (ctl-exo), chronic morphine-treated astrocyte-derived exosomes (mor-exo) significantly increased the levels of cAMP and intracellular Ca^2+^ and activated CAMKII and CREB in morphine-treated SH-SY5Y cells. It is suggested that morphine-activated astrocytes could exacerbate the morphine-dependence-like effects of neuronal SH-SY5Y cells by exosomes. Although mor-exo showed no significant effect on the activation of NMDAR1 receptors in SH-SY5Y cells, mor-exo still upregulated the activation of CAMKII and CREB. The increase of intracellular Ca^2+^ may contribute to the activation of CAMKII and its downstream protein, CREB. However, whether astrocyte-derived exosomes shuttled Ca^2+^ into recipient neuronal SH-SY5Y cells and caused the increase of intracellular Ca^2+^ levelsremains to be examined in further study. Sinomenine-pretreated astrocyte-derived exosomes (Sino-exo) significantly decreased the levels of cAMP and intracellular Ca^2+^ and inhibited the activation of CAMKII and CREB. This study showed that activated astrocytes may regulate the function of recipient neuronal SH-SY5Y cells through exosomes and participate in the formation and maintenance of morphine dependence through the CAMKII–CREB pathway. Fortunately, sinomenine inhibited the activation of astrocytes induced by morphine. Furthermore, sinomenine-pretreated astrocyte-derived exosomes attenuated the morphine-dependence-like effects of SH-SY5Y cells. However, whether the mechanism is related to the changes in components within the exosomes and whether the astrocyte-derived exosomes have the same function in morphine-dependent animals need to be further explored in future studies.

## 4. Materials and Methods

### 4.1. Reagents and Assay Kits

Sinomenine (No. 2000058, purity 94.9%) was bought from Hunan Zhengqing Pharmaceutical Co., Ltd., China. Morphine (30 mg/mL) was obtained from the drug supply station of the People’s Liberation Army, China. Naloxone, NMDA, and PKH67 were purchased from Sigma-Aldrich (St. Louis, MO, USA). Phospho-NMDAR1 (Ser897), NMDAR1, p-CAMKII (Ser286), CAMKII, and p-CREB (Ser133) antibodies were purchased from Affinity Biosciences (Cincinnati, OH, USA). CREB antibody and anti-rabbit IgG antibodies were purchased from Cell Signaling Technology (Boston, MA, USA). Calcium fluorescent probe Fluo-8 AM, GFAP, TSG101, CD81, and CD63 antibodies were bought from Abcam (Cambridge, UK). Cyclic-AMP direct immunoassay kit (colorimetric) was obtained from BioVision (Milpitas, CA, USA). The chemiluminescence reaction kit (ECL) was purchased from Thermo Fisher Scientific (Waltham, MA, USA).

### 4.2. Animals

Kunming mice (male, 18–22 g, aged 5–6 weeks) were obtained from the Experimental Animal Center of Southern Medical University (License key: SCXY2016-0167). Mice were acclimatized in a controlled specific pathogen free (SPF) environment (20–22 °C, 55–60% humidity, 12 h light/dark cycle, ad libitum access to food and water). All procedures involving animals were approved by the Ethics Committee on the Use and Care of Animals, Southern Medical University, China.

### 4.3. Establishment of a Morphine-Dependence Model by Conditioned Place Preference (CPP), and Treatment Details

#### 4.3.1. Apparatus

The CPP apparatus used was similar to that used in our previous studies [37]. Briefly, the CPP apparatus was divided into two equally sized compartments by a mobile partition. The black compartment was painted black with a smooth bottom, while the white compartment was painted white with a rough bottom. Mice could move freely between the two compartments when the mobile partition was removed.

#### 4.3.2. CPP Phases

The CPP consisted of 3 phases, namely preconditioning, conditioning, and postconditioning phases (Figure 1A).

#### 4.3.3. Preconditioning Phase

In the preconditioning phase (day 1–3), mice were freed into the CPP apparatus for 15 min each day. On day 3, the amount of time each mouse spent on each compartment (the white and the black compartment) within 15 min in the CPP apparatus was recorded as the preconditioning time. The mice that showed natural preference for the white compartment were excluded from further study. The remaining mice were divided into 4 groups (*n* = 8): control, morphine (9 mg/kg), morphine + Sino (60 mg/kg), and morphine + Sino (60 mg/kg) + NMDA (10 mg/kg).

#### 4.3.4. Conditioning Phase

In the conditioning phase (day 4–9), at 09:00 each day, mice from the control group received a sterile 0.9% physiological saline injection (10 mL/kg, subcutaneous injection, s.c.), whereas mice from the other 3 groups received a morphine injection (9 mg/kg, s.c.). The mice were immediately placed in the white compartment for 45 min. After an interval of 8 h, at 17:00, the mice from all groups received a sterile 0.9% physiological saline injection (10 mL/kg, s.c.) and were immediately placed in the black compartment for 45 min. From day 7 to 9, the mice from the morphine + Sino group received a daily injection of sinomenine solution (60 mg/kg, intraperitoneal injection, i.p.) 45 min before morphine injection, whereas mice from the morphine + Sino + NMDA group received a daily injection of sinomenine solution (60 mg/kg, i.p.) and NMDA solution (10 mg/kg, i.p.) 45 min before morphine injection.

#### 4.3.5. Postconditioning Phase

Twenty-four hours after the last injection of morphine (day 10), the mice from all groups were freed into the CPP apparatus. The amount of time each mouse spent in the white compartment within 15 min in the CPP apparatus was recorded as the postconditioning time. Afterwards, all mice were sacrificed for brain tissue collection.

### 4.4. Immunohistochemistry Staining

Brain tissues were fixed in 4% paraformaldehyde for 72 h, dehydrated in graded ethanol, embedded in paraffin, and cut into 5-μm-thick slices. Immunohistochemistry staining was performed on the tissue slices following procedures described in a previous study [38]. Primary antibodies (p-NMDAR1 1:250, NMDAR1 1:250, GFAP 1:250) were diluted in 5% skim milk.

### 4.5. Western Blotting

Protein samples were electrophoresed on 5–10% sodium dodecyl sulfate (SDS)-polyacrylamide gels and transferred to a polyvinylidene fluoride (PVDF) membrane. The membrane was blocked with 5% skim milk (diluted in 0.1% TBST) for 1 h at 25 °C. The membrane was incubated with primary antibody (p-NMDAR1 1:500, NMDAR1 1:500, p-CAMKII 1:500, CAMKII 1:500, p-CREB 1:500, CREB 1:500, GFAP 1:500, TSG101 1:1000, CD81 1:1000, CD63 1:1000) overnight at 4 °C and incubated with secondary antibodies for 1 h at 25 °C. Immunoreactive proteins were detected using enhanced chemiluminescence (ECL) reagents.

### 4.6. Cell Culture

The human neuroblastoma cell line SH-SY5Y was obtained from the ATCC (Manassas, VA, USA). Cells were cultured in RPMI-1640 medium containing 10% (*v*/*v*) fetal bovine serum (Gemini) and 0.5% (*v*/*v*) penicillin/streptomycin (Gibco) and incubated at 37 °C and 5% CO_2_.

Rat primary astrocytes were prepared according to a previous study’s method with slight modifications [39]. Cells were collected from the brain cortex tissues of neonatal Sprague-Dawley rats (aged 1–2 days) and seeded onto cell culture flasks. Cells were cultured in DMEM/F12 medium containing 10% fetal bovine serum and 0.5% penicillin/streptomycin and incubated at 37 °C and 5% CO_2_. Once the cells reached confluence, they were shaken for 18 h at 37 °C, passaged by trypsinization, and seeded onto cell culture plates. After 2 passages, the astrocytes were used for further experiments. Immunocytochemical analysis showed that the cultured cells were comprised of over 95% GFAP-positive astrocytes (see Figure S1 in the Supplementary Materials).

### 4.7. Determination of cAMP Level in SH-SY5YCells

SH-SY5Y cells were incubated with 1 mL 0.1 M HCl for every 35 cm^2^ surface area at 25 °C for 20 min and then scraped off the surface. The cell samples were collected by centrifugation at 15,294× *g* for 10 min. The level of cAMP was determined by using a commercial assay kit (BioVision, CA, USA).

### 4.8. Determination of Intracellular Calcium ([Ca^2+^]) in SH-SY5Y Cells

Cells were suspended in cell culture medium, loaded with 4 μM Fluo-8 AM, and incubated for 45 min at 37 °C and 5% CO_2_. Afterwards, the cells were collected by centrifugation at 115× *g* for 5 min and washed twice with HHBS. The cell samples were suspended in 300 μL HHBS and detected by flow cytometry (excitation at 490 nm, emission at 530 nm).

### 4.9. Extraction of Astrocyte-Derived Exosomes by Differential Centrifugation

Cultured rat primary astrocytes were treated with PBS, morphine (100 μM), or morphine and sinomenine (200 μM) and cultured for 48 h in DMEM/F12 medium containing 5% exosome-depleted FBS. The exosome-depleted FBS was previously ultracentrifuged for 14 h at 120,000× *g* to remove exosomes in FBS. Exosomes were extracted from these cell culture mediums by differential centrifugation as previously described [40].

### 4.10. Transmission Electron Microscopy (TEM)

Exosomes pellets were suspended in PBS for negative staining. Twenty microliters of exosomes were placed onto carbon-coated copper grids and allowed to absorb for 2 min. The copper grids were washed by distilled deionized water and then allowed to semidry for 15–20 min at 25 °C before observation in TEM (Hitachi H7650 TEM, Tokyo, Japan).

### 4.11. Nanoparticle Tracking Analysis 

A Zetaview Particle Metrix (Software ZetaView 8.04.02, Inning am Ammersee, Germany) was used to assess the size of the exosomes suspended in PBS. The extracted exosomes were diluted with 1× PBS at a ratio of 1:25,000 and injected into the Zetaview instrument for nanoparticle tracking analysis (NTA) to assess the size distribution and concentration of the exosomes.

### 4.12. Exosome Labeling by Fluorescent Dye PKH67

Exosomes diluted in 250 μL PBS were added to 1 mL Diluent C. In parallel, 4 μL PKH67 was added to 1 mL Diluent C and incubated with the exosome solution for 4 min at 25 °C. Two milliliters of 1% fetal bovine serum (BSA) was added to the solution to bind excess dye for 1 min at 25 °C. The mixture containing labeled exosomes wascentrifuged at 100,000× *g* for 70 min and the exosome pellet was suspended in 1 × PBS. For the evaluation of exosome uptake, the labeled exosomes were diluted in RPMI-1640 medium containing 5% exosome-depleted FBS and incubated with SH-SY5Y cells for 12 h at 37 °C and 5% CO_2_. After incubation, the culture medium was discarded and SH-SY5Y cells were washed 3 times with PBS. The cells were stained with DAPI, incubated for 10 min at 25 °C, and imaged with an inverted fluorescence microscope (Olympus, Tokyo, Japan).

### 4.13. Statistical Analysis

Statistical analyses were performed using Graph Pad Prism 5.0 (Pad Prism, La Jolla, CA, USA). All data were expressed as the mean ± SD. One-way analysis of variance and Student’s *t*-test were used for the determination of significant differences in measurements between groups, in which *p* < 0.05 was considered significant.

## 5. Conclusions

In conclusion, sinomenine had an inhibitory effect on morphine dependence in vitro and in vivo. For the first time, we discovered that its mechanism may be related to the inhibition of the NMDAR1/CAMKII/CREB pathway. Furthermore, we found that morphine-treated astrocytes aggravated morphine-dependence-like effects in recipient neuronal SH-SY5Y cells by exosomes; these effects were attenuated by sinomenine, which altered the function of these astrocyte-derived exosomes. This may be one mechanism by which sinomenine prevents morphine dependence, which provides a new experimental basis for preventing drug dependence using traditional Chinese medicine.

## Supplementary Materials

Supplementary materials can be found online.

## Abbreviations

Sino	sinomenine
mor	morphine
nal	naloxone
exo	exosomes
NMDAR1	*N*-methyl-d-aspartate receptor 1
CAMKII	Ca^2+^/calmodulin-dependent protein kinase II
CREB	cyclic adenosine monophosphate response element-binding protein
