# Supplementary material for: Sinomenine Protects Against Morphine Dependence through the NMDAR1/CAMKII/CREB Pathway: A Possible Role of Astrocyte-Derived Exosomes
Source: Molecules. 2018 Sep 17;23(9):2370. doi: 10.3390/molecules23092370 (PMC6225372; doi:10.3390/molecules23092370)
Supplement: Supplementary file 1 [file molecules-23-02370-s001.zip › molecules-350429-SI.pdf]

Sinomenine Protects Against Morphine Dependence through the NMDAR1/CAMKII/CREB Pathway: A Possible Role of Astrocyte-Derived Exosomes

Supplementary Materials:

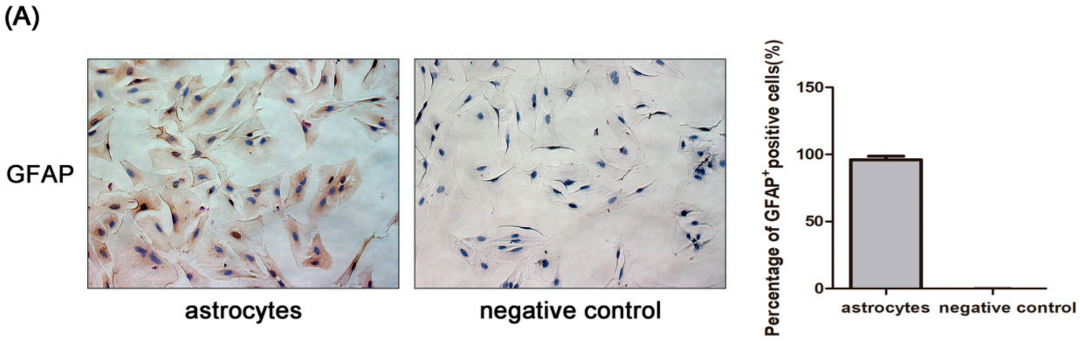

Figure S1. Purified cultured primary astrocytes. Immunocytochemical analysis showed that the cultured cells comprised of over 95% GFAP-positive astrocytes (n=3).

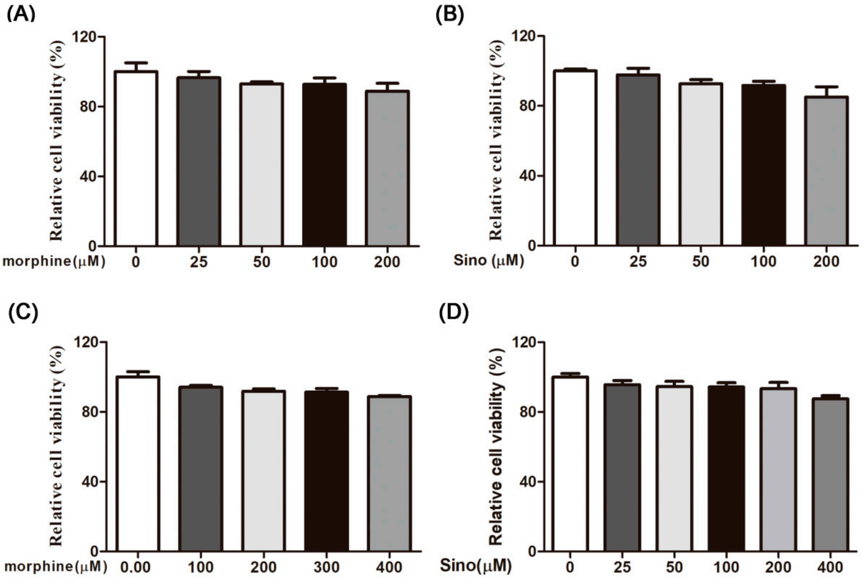

Figure S2. Relative cell viability detected by MTT assay. (A) The effect of morphine on the relative cell viability of SH-SY5Y cells for 48h (n=3). The highest dose at 100 μM did not show significant cytotoxicity in SH-SY5Y cells (cell viability > 90%). (B) The effect of sinomenine on the relative cell viability of SH-SY5Y cells for 24h (n=3). The highest dose at 100 μM did not show significant cytotoxicity in SH-SY5Y cells (cell viability > 90%). (C) The effect of morphine on the relative cell viability of cultured primary astrocytes for 48h (n=3). The concentration at 100 - 300 μM did not show significant cytotoxicity in cultured primary astrocytes (cell viability > 90%). (D) The effect of sinomenine on the relative cell viability of cultured primary astrocytes for 48h (n=3). The concentration at 200 μM did not show significant cytotoxicity in SH-SY5Y cells (cell viability > 90%).
